# Nuclear–Electron Correlation Effects and Their Photoelectron Imprint in Molecular XUV Ionisation

**DOI:** 10.3389/fchem.2022.942633

**Published:** 2022-08-05

**Authors:** Karl Michael Ziems, Jakob Bruhnke, Volker Engel, Stefanie Gräfe

**Affiliations:** ^1^ Institute of Physical Chemistry, Friedrich Schiller University Jena, Jena, Germany; ^2^ Max Planck School of Photonics, Jena, Germany; ^3^ Institut für Physikalische und Theoretische Chemie, Universität Würzburg, Würzburg, Germany; ^4^ Abbe Center of Photonics, Friedrich Schiller University, Jena, Germany; ^5^ Fraunhofer Institute for Applied Optics and Precision Engineering, Jena, Germany

**Keywords:** ultrafast phenomena, XUV, attosecond dynamics, photoionisation, TDSE, correlation effects, entanglement, photoelectron spectrum

## Abstract

The ionisation of molecules by attosecond XUV pulses is accompanied by complex correlated dynamics, such as the creation of coherent electron wave packets in the parent ion, their interplay with nuclear wave packets, and a correlated photoelectron moving in a multi-centred potential. Additionally, these processes are influenced by the dynamics prior to and during the ionisation. To fully understand and subsequently control the ionisation process on different time scales, a profound understanding of electron and nuclear correlation is needed. Here, we investigate the effect of nuclear–electron correlation in a correlated two-electron and one-nucleus quantum model system. Solving the time-dependent Schrödinger equation allows to monitor the correlation impact pre, during, and post-XUV ionisation. We show how an initial nuclear wave packet displaced from equilibrium influences the post-ionisation dynamics by means of momentum conservation between the target and parent ion, whilst the attosecond electron population remains largely unaffected. We calculate time-resolved photoelectron spectra and their asymmetries and demonstrate how the coupled electron–nuclear dynamics are imprinted on top of electron–electron correlation on the photoelectron properties. Finally, our findings give guidelines towards when correlation resulting effects have to be incorporated and in which instances the exact correlation treatment can be neglected.

## 1 Introduction

The fact that, if formerly non-interacting particles have interacted at some time, their wave function can no longer be expressed in a simple product form (Blum, 2012), has far-reaching consequences in many particle systems. For example, this situation appears in electronic structure calculations and there is termed electron–electron correlation (Kutzelnigg, 1994). The latter determines—to a great deal—the structure and behaviour of matter. In the field of quantum information, this correlation effect is associated with the entanglement of particles (Nielson and Chuang, 2000; Horodecki et al., 2009). As for molecules, not only electron–electron but also electron–nuclear and nuclear–nuclear interactions are of importance. Here, nuclear geometry deformations, in general, lead to the modification of the electronic density, which is responsible for chemical bonding.

The interaction of molecules with strong and ultrashort laser pulses leads, besides many other strong-field phenomena (Wolter et al., 2015; Pukhov, 2002; Krausz and Ivanov, 2009; Corkum and Krausz, 2007; Joachain et al., 2012), to single or multiple ionisation. A single XUV pulse is able to directly produce photoelectrons with different kinetic energies. With respect to the particle correlations mentioned previously, several questions arise where some of these are: What does a coupled electronic–nuclear motion look like during and after the ionisation process? Can features appearing in photoelectron spectra be related to electron–electron and electron–nuclear correlation? What characteristics appear in the post-ionisation dynamics of the charged particles?

Such fundamental issues will be taken up in the present work. Naturally, regarding the complexity of a molecule possessing many electronic and nuclear degrees of freedom, a complete quantum description of a field-triggered ionisation is simply out of reach today. One may then search for physically reasonable models to address the questions posed. It should be clear that they have to go beyond single active electron approximations and include the motion of the nuclei, most desirably on the same level as the electrons. In an early study, Lein et al. (2002) studied the single and double ionisation of the hydrogen molecule involving the motion of all particles in a single dimension and Sukiasyan et al. (2012) described the one-electron photoionisation for a 1D-Helium atom with two active electrons. To understand the impact of ionisation on the parent ion dynamics in real molecules, approximated quantum chemical methods neglecting the explicit ionisation pump can be applied and are powerful tools to unravel electron dynamics post ionisation (Ayuso et al., 2017; Nisoli et al., 2017) and study the nuclear decoherence effect on electronic wave packets (Vacher et al., 2015, 2017).

A useful model to study electron–nuclear correlation effects is the so-called Shin–Metiu model (Shin and Metiu, 1995, 1996). It consists of an electron and a nucleus that move in one dimension in an additional field of two positive charges. Originally devised to describe charge-transfer processes, it was later used to illustrate features of, e.g., coupled electronic–nuclear quantum (Hader et al., 2017; Albert et al., 2017; Schaupp and Engel, 2019b, 2022) and classical dynamics (Schaupp and Engel, 2019a) or two-dimensional coherent femtosecond spectroscopy (Albert et al., 2015). The model was also used to study photoionisation (Falge et al., 2011, 2012a, 2017).

The simple Shin–Metiu model was later extended to include the motion of a second electron, which made it possible to introduce time-dependent electron localisation functions (ELF) and characterise the influence of nuclear motion on these (Erdmann et al., 2004). Also, the wave-packet dynamics in spin-coupled electronic states could be described (Falge et al., 2012b).

Here, we extend our work on XUV ionisation in a molecular model system comprised of fully correlated two electrons and one nucleus (Fröbel et al., 2020). We study the impact of electron–nuclear correlation upon electron–electron correlation on the complete XUV ionisation process monitoring the influence pre, during, and post ionisation. Consequently, we dissect the effects on the parent ion, as well as on the photoelectron. Finally, we report on an imprint of the two-electron correlated bound dynamics on the photoelectron spectrum’s asymmetry, thus yielding an observable to measure the electrons’ density behaviour caused by nuclear correlation. This is a natural extension of our former work limited to a single active electron system (Falge et al., 2012a, 2017) and shows that the concept also holds for more complex systems. Moreover, by thoroughly dissecting the different effects present in a full quantum dynamical study with correlated particles, we provide guidance for future investigations resting on more approximated methods.

This study is organised as follows: In Section 2, we briefly introduce the model system, its potential energy surface, the numerical details for solving the time-dependent Schrödinger equation (TDSE), and different analysis tools. In presenting our results, we start by introducing the laser-free non-equilibrium dynamics and, subsequently, report on the impact of correlation effects on 1) the attosecond electron dynamics during ionisation, 2) the post-ionisation dynamics in the parent ions, and 3) the photoelectron. In the last section, we discuss how the asymmetry of the integrated photoelectron spectra shows imprints of resonance dependencies into the continuum and the coupled electron–nuclear dynamics.

## 2 Theoretical Background

In the following, we briefly describe the model system and numerical procedure. For more details, we refer to our recent work (Fröbel et al., 2020), where we introduce the model in the context of ionisation. Atomic units are used throughout the study.

### 2.1 Molecular Model System

#### 2.1.1 Full Three-Dimensional Model

**FIGURE 1:**
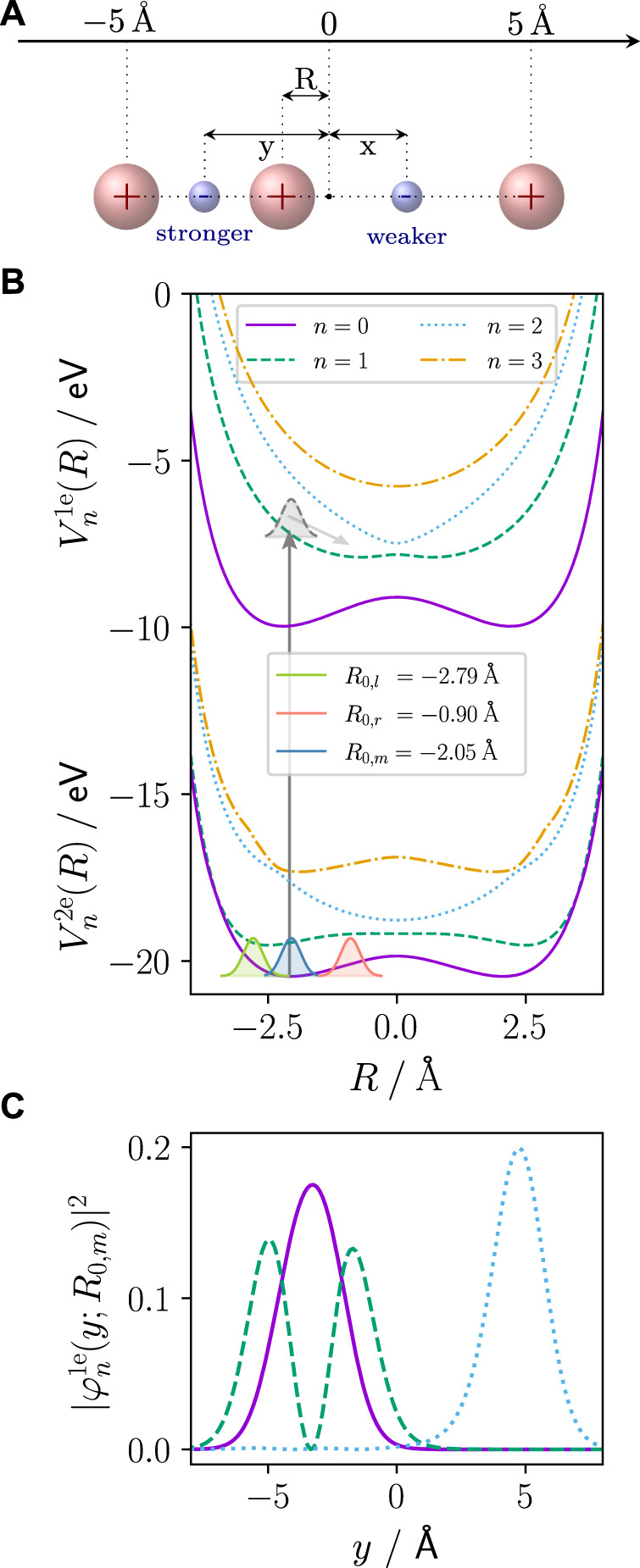
**(A)** Extended Shin–Metiu model system: two electrons (*x*, *y*) and one nucleus I move in one dimension in the field of the two outer fixed (point charge) nuclei (*R*). For *R* < 0 as in the focus of investigation here, the left electron (here: *y*) is stronger bound, while the right electron (here: *x*) is weaker bound. The electrons are indistinguishable and are just given defined labels here for visualisation purposes. **(B)** PECs of the 2e system, $V_{n}^{2\text{e}}\left(R\right)$ , and the 1e parent ion, $V_{n}^{1\text{e}}\left(R\right)$ . The vertical line indicates one-photon ionisation and the population of the parent ion states. As an example, the nuclear wave packet in the *n* = 1 ion state is shown together with the gradient exerted by the PEC on it (grey shaded Gaussian). In the 2e system ground state (*n* = 0), the three different initial nuclear wave packets investigated in this work are shown. The nuclear wave packet near the equilibrium position of *R*_0,*m*_ = −2.05 Å (blue shaded Gaussian), and the two non-equilibrium starting wave packets starting at the isopotential turning points left, *R*_0,*l*_ = −2.79 Å (green shaded Gaussian), and right, *R*_0,*r*_ = −0.90 Å (red shaded Gaussian), of the equilibrium. **(C)** The absolute square of the first three 1e parent ion wave functions shows that *n* = 2 (blue dotted) is located at *y* > 0 (weaker bound).

To capture electron–nuclear and electron–electron correlation in molecular XUV ionisation, we use the one-dimensional extended Shin–Metiu model system, which includes two electrons (*x*, *y*) and a central nucleus with coordinate *R* (Shin and Metiu, 1995, 1996; Erdmann et al., 2004). Furthermore, two fixed nuclear point charges (*Z*_1_, *Z*_2_) at ± *L*/2 define the outer potential barriers. The particle configuration is sketched in Figure 1A. The molecular Hamiltonian reads:

$$H_{0}=\frac{{\hat{P}}^{2}}{2M}+\frac{{\hat{P}}_{x}^{2}}{2}+\frac{{\hat{P}}_{y}^{2}}{2}+{\hat{V}}^{2\text{e}}\left(x,y,R\right),$$ (1)

where *M* is the nuclear mass, $\hat{P}$ the nuclear momentum operator, and ${\hat{p}}_{x},{\hat{p}}_{y}$ the electron momentum operators. The potential is defined as:

$$\begin{array}{ll} {\hat{V}}^{2\text{e}}\left(x,y,R\right)= & \frac{Z_{1}Z}{\left|L/2-R\right|}+\frac{Z_{2}Z}{\left|L/2+R\right|}-\frac{Z\;\text{erf}\left(|R-y|/R_{c}\right)}{\left|R-y\right|}\\ & -\frac{Z_{1}\;\text{erf}\left(|L/2-y|/R_{f}\right)}{\left|L/2-y\right|}-\frac{Z_{2}\;\text{erf}\left(|L/2+y|/R_{f}\right)}{\left|L/2+y\right|}\\ & -\frac{Z\;\text{erf}\left(|R-x|/R_{c}\right)}{\left|R-x\right|}-\frac{Z_{1}\;\text{erf}\left(|L/2-x|/R_{f}\right)}{\left|L/2-x\right|}\\ & -\frac{Z_{2}\;\text{erf}\left(|L/2+x|/R_{f}\right)}{\left|L/2+x\right|}+\frac{\text{erf}\left(|x-y|/R_{e}\right)}{\left|x-y\right|}. \end{array}$$ (2)

Here, we set the charges *Z*_1_ = *Z*_2_ = *Z* = 1, *M* to the proton mass, the screening parameters *R*_*c*_ = *R*_*f*_ = *R*_*e*_ = 1.5 Å and *L* = 10 Å for the outer point charges. The potential contains soft Coulomb interactions between the moving particles, parameterised by error functions (erf). The model mimics a generic molecular system leading to a qualitative description of processes. It does not represent a specific class of molecules, such as linear molecules, especially since the central moving nucleus has unscreened interactions with the outer fixed nuclei preventing dissociation. Such effect would be essential for strong-field interactions with seemingly similar linear triatomic systems, but they are not in the scope of this work. Moreover, we are restricted to one nuclear degree of freedom, thus, not investigating nuclear–nuclear correlation. Since the model is one-dimensional, effects of the orbital angular momentum of the electrons are not regarded. We note that the system is already of ionic type. Nevertheless, in what follows, we will refer to the removal of an electron by the external field as an ionisation process.

While the reduced dimensionality of the model allows for solving the dynamics of all three particles, for interpretation, it is useful to obtain the electronic eigenstates and the potential energy curves (PECs) of the two-electron (2e) system. Therefore, we solve the time-independent electronic Schrödinger equation:

$$\left[\frac{{\hat{P}}_{x}^{2}}{2}+\frac{{\hat{P}}_{y}^{2}}{2}+{\hat{V}}^{2\text{e}}\left(x,y,R\right)\right]\varphi_{n}^{2\text{e}}\left(x,y;R\right)=V_{n}^{2\text{e}}\left(R\right)\;\varphi_{n}^{2\text{e}}\left(x,y;R\right).$$ (3)

where ${\hat{p}}_{x}$ and ${\hat{p}}_{y}$ are the electronic momentum operators. This yields the adiabatic electronic eigenstates $\varphi_{n}^{2\text{e}}\left(x,y;R\right)$ and the potentials $V_{n}^{2\text{e}}\left(R\right)$ . All our calculations are restricted to the singlet case, i.e., the appearing wave functions are symmetric upon exchanging *x* and *y*. Upon removal of an electron through the XUV interaction, the remaining one-electron (1e) parent ion system has the potential:

$$\begin{array}{ll} {\hat{V}}^{1\text{e}}\left(y,R\right)= & \frac{Z_{1}Z}{\left|L/2-R\right|}+\frac{Z_{2}Z}{\left|L/2+R\right|}-\frac{Z\;\text{erf}\left(|R-y|/R_{c}\right)}{\left|R-y\right|}\\ & -\frac{Z_{1}\;\text{erf}\left(|L/2-y|/R_{f}\right)}{\left|L/2-y\right|}-\frac{Z_{2}\;\text{erf}\left(|L/2+y|/R_{f}\right)}{\left|L/2+y\right|}. \end{array}$$ (4)

The respective electronic eigenstates $\left(\varphi_{n}^{1\text{e}}\left(y;R\right)\right)$ and the PECs $\left(V_{n}^{1\text{e}}\left(R\right)\right)$ are determined by the time-independent electronic Schrödinger equation

$$\left[\frac{{\hat{p}}_{y}^{2}}{2}+{\hat{V}}^{1\text{e}}\left(y,R\right)\right]\varphi_{n}^{1\text{e}}\left(y;R\right)=V_{n}^{1\text{e}}\left(R\right)\;\varphi_{n}^{1\text{e}}\left(y;R\right).$$ (5)

The PECs of both the 2e and 1e systems are shown in Figure 1B for the lowest four electronic eigenstates. It is important to point out that for the 1e system (parent ion), and *R* < 0 (which is the case throughout this work), the electron resides either left, at negative *y* values (stronger bound), or right, at positive *y* values (weaker bound), of the central nucleus depending on its electronic state. For the *n* = 2 1e state, the electronic eigenfunction’s probability density, $|\varphi_{2}^{1\text{e}}\left(y;R\right){|}^{2}$ , is located at the weaker bound site. The other 1e states shown in Figure 1C are located left of the central nucleus (stronger bound site).

We define the initial wave function as the product of the 2e adiabatic ground state (*n* = 0) and a Gaussian-shaped vibrational wave packet, *χ*(*R*):

$$\chi \left(R\right)=N_{0}\;e^{-\beta_{R}{\left(R-R_{0,l/m/r}\right)}^{2}},$$ (6)

$$\Psi \left(x,y,R,t_{0}\right)=\varphi_{0}^{2\text{e}}\left(x,y;R\right)\;\chi \left(R\right),$$ (7)

with the normalisation constant *N*_0_ and *β*_*R*_ = 7.14 Å^−2^. As shown in Figure 1B, we regard three different initial vibrational wave packets, which differ with respect to the centre of the Gaussian in Eq. 6. In particular, we use the equilibrium configuration *R*_0,*m*_ = −2.05 Å (blue shaded Gaussian), and two non-equilibrium configurations, where one is placed to the left *R*_0,*l*_ = −2.79 Å (green shaded Gaussian) and the other to the right *R*_0,*r*_ = −0.90 Å (red shaded Gaussian) of *R*_0,*m*_. The two non-equilibrium positions were chosen isopotentially.

The system interacts with a linearly polarised XUV pulse defined via its vector potential, A(t) , with polarisation aligned along with the molecular axis of the model:

$$A\left(t\right)=\frac{E_{0}}{\omega }\;g\left(t+t_{0}\right)\;\sin\left(\omega \left(t+t_{0}\right)\right).$$ (8)

We use an electric field strength of *E*_0_ = 0.169 a.u. (corresponding to an intensity of *I* = 10^15^ W/cm^2^), an angular frequency of *ω* = 0.570 a.u. (*λ* = 80 nm = 15.5 eV), and a full-width at half-maximum (FWHM) of *τ* = 5 fs for the Gaussian pulse envelope function *g*(*t*). The comparatively long FWHM was chosen to avoid possible few-cycle effects leading to pulse-dependent intrinsic asymmetries in the photoelectron spectrum (PES). For the parameters chosen here, the light pulse does not influence the asymmetry of the PES, and despite the high field strength, the simple one-photon picture of energy conservation between light pulse, parent ion, and photoelectron holds. The different pulse interaction times *t*_0_, for different simulation setups are discussed and introduced as follows: the propagation starts at *t* = *t*_0_ − 2*τ*. The full time-dependent Hamiltonian in velocity gauge and dipole approximation reads:

$$H\left(t\right)=\frac{{\hat{P}}^{2}}{2M}+\frac{{\hat{p}}_{x}^{2}}{2}+\frac{{\hat{p}}_{y}^{2}}{2}+{\hat{V}}^{2\text{e}}\left(x,y,R\right)+e\;A\left(t\right)\left(-\frac{\hat{P}}{M}+{\hat{p}}_{x}+{\hat{p}}_{y}\right),$$ (9)

#### 2.1.2 Approximations: Frozen and Single Point Charge Nucleus

In order to understand the role of the nuclear degree-of-freedom in the quantum dynamical simulations, we compare the complete electron–nuclear dynamics to the case of *1*) a frozen nuclear wave packet and *2*) a single point charge calculation. In the frozen nuclear wave packet approximation, *1*), the nuclear dimension becomes parametric and is only used to sample the nuclear wave packet on the grid by several 2D simulations of the electronic degrees of freedom. Hence, the Hamiltonian, Eq. 9, is missing the nuclear kinetic energy and XUV interaction term. The *R*-dimension in the potential and the wave function becomes parametric. The single point calculation, *2*), completely neglects the wave packet nature of the central nucleus and treats the central nucleus as a point charge at a fixed position leading to a single 2D simulation of the electronic degrees of freedom. This leaves the Hamiltonian of Eq. 9 without any explicit or parametric *R* dependence, yielding a two-dimensional wave function depending on *x*, *y*.

### 2.2 Numerical Details

The time-dependent Schrödinger equation is as follows:

$$i\frac{\partial }{\partial t}\Psi \left(x,y,R,t\right)=H\left(t\right)\Psi \left(x,y,R,t\right),$$ (10)

with the Hamiltonian defined in Eq. 9 is solved numerically on a grid of dimensions [ − 240, 240]Å with 1,024 grid points for *x* and *y* (electronic dimensions) and [ − 4.99, 4.99]Å with 128 grid points for *R* (nuclear dimension). The integration is performed with a time step of 5as using the split-operator technique (Feit et al., 1982) and the FFTW three libraries (Frigo and Johnson, 1998) for Fourier transforms. This setup is used for all calculations unless stated otherwise. The time-independent 2e and 1e Schrödinger equations defined in Eqs. (3) and (5), respectively, are numerically solved with the relaxation method, solving the TDSE in imaginary time (Kosloff and Tal-Ezer, 1986).

To avoid grid reflection, cut-off functions are applied each time step to the full wave function in the asymptotic region of the molecular potential

$$f\left(x,y\right)={\left[1+e^{\zeta_{1}\;\left(|x|-\zeta_{2}\right)}\right]}^{-1}\;{\left[1+e^{\zeta_{1}\;\left(|y|-\zeta_{2}\right)}\right]}^{-1}$$ (11)

with the parameters *ζ*_1_ = 0.085 a.u. and *ζ*_2_ = 492 a.u. (Heather and Metiu, 1987).

In the following, we introduce three analysis tools of the full-wave function, Ψ(*x*, *y*, *R*, *t*), in order to arrive at a deeper understanding of the ionisation dynamics. The exact ionised wave function comprised of having one electron in the continuum, whilst the other electron is still bound in the parent ion is unknown for such molecular, many-particle, and multi-centred systems. The following approaches circumvent this problem by using grid-based functions and projection operators.

#### 2.2.1 Outer Wave Functions, $\Psi_{\text{out}}^{\text{fwd/bwd}}\left(p_{x},y,R,t\right)$ , Long-Time Limit

To obtain the part of the wave function representing the ionised system at long times, the outgoing parts of the wave function in *x* direction are collected. Since the wave function is fully mirror symmetric in *x* and *y*, we arbitrarily choose *x* as a dimension of ionisation, while *y* characterises the bound electron in the parent ion. To this end, we define a mask function in forward (fwd), *x* > 0, and backward (bwd), *x* < 0, direction using the same values of *ζ*_1_, *ζ*_2_ as mentioned previously.

$${\tilde{f}}^{\text{fwd}}\left(x,y\right)=\left(1-{\left[1+e^{\zeta_{1}\;\left(x-\zeta_{2}\right)}\right]}^{-1}\right)\Theta \left(25\;\overset{{}^{\circ}}{A}-|y|\right),$$ (12)

$${\tilde{f}}^{\text{bwd}}\left(x,y\right)=\left(1-{\left[1+e^{\zeta_{1}\;\left(-x-\zeta_{2}\right)}\right]}^{-1}\right)\Theta \left(25\;\overset{{}^{\circ}}{A}-|y|\right),$$ (13)

where the Heaviside function restricts the outer wave function to grid values of −25 Å <y<25 Å, thus, neglecting double ionisation. At each time step, ${\tilde{f}}^{\text{fwd/bwd}}\left(x,y\right)$ is applied to the total wave function, Fourier-transformed (FT) with respect to the electronic coordinate *x* and added coherently to the parts already localised in the outer regions in order to yield the outer wave functions:

$$\Psi_{\text{out}}^{\text{fwd/bwd}}\left(p_{x},y,R,t\right)=\Psi_{\text{out}}^{\text{fwd/bwd}}\left(p_{x},y,R,t-\Delta t\right)+{FT}_{x}\left[{\tilde{f}}^{\text{fwd/bwd}}\left(x,y\right)\Psi \left(x,y,R,t\right)\right].$$ (14)

Consequently, $\Psi_{\text{out}}^{\text{fwd/bwd}}\left(p_{x},y,R,t\right)$ is only propagated in momentum space in *x* dimension. The outer wave functions are used to calculate the PESs, state resolved to particular 1e states in the parent ion, $\sigma_{n}^{\text{fwd/bwd}}\left(p_{x}\right)$ , and to obtain the total integrated asymmetry *A*. The former is obtained by projection on the adiabatic 1e states at the end of the time propagation, in the limit *t* → *∞*

$$\sigma_{n}^{\text{fwd/bwd}}\left(p_{x}\right)=\int {\left|\int \varphi_{n}^{1\text{e}}\left(y;R\right)\Psi_{\text{out}}^{\text{fwd/bwd}}\left(p_{x},y,R,t\to \infty \right)\;dy\right|}^{2}\;dR.$$ (15)

The total integrated asymmetry of the PES is calculated as

$$A=\underset{n}{\sum }\int \frac{\sigma_{n}^{\text{fwd}}\left(p_{x}\right)-\sigma_{n}^{\text{bwd}}\left(p_{x}\right)}{\sigma_{n}^{\text{fwd}}\left(p_{x}\right)+\sigma_{n}^{\text{bwd}}\left(p_{x}\right)}dp_{x}.$$ (16)

#### 2.2.2 Channel Wave Functions, $\Psi_{\text{ch}}^{\text{fwd/bwd}}\left(x,y,R,t\right)$ , Intermediate Times

In order to investigate the ionised system at intermediate times in fwd and bwd directions, we define the channel wave functions.

$$\Psi_{\text{ch}}^{\text{fwd}}\left(x,y,R,t\right)=\Theta \left(-25\;\overset{{}^{\circ}}{A}+x\right)\Theta \left(25\;\overset{{}^{\circ}}{A}-|y|\right)\Psi \left(x,y,R,t\right),$$ (17)

$$\Psi_{\text{ch}}^{\text{bwd}}\left(x,y,R,t\right)=\Theta \left(-25\;\overset{{}^{\circ}}{A}-x\right)\Theta \left(25\;\overset{{}^{\circ}}{A}-|y|\right)\Psi \left(x,y,R,t\right).$$ (18)

Again, these wave functions represent the ionised system and monitor the parent ion electron (*y*) located in the grid range −25 Å <y<25 Å, while the photoelectron (*x*) is at larger position values on the grid, |*x*| > 25 Å.

#### 2.2.3 Bound/Continuum Wave Function, Ψ_1b/1c_ (*x*, *y*, *R*, *t*), Early Times

Additionally, to understand the attosecond electron dynamics at early times during XUV pulse interaction, the exact 1e-bound/1e-continuum (1b/1c) wave function is needed. For this, we define a bound/continuum wave function, i.e., 1e-bound/1e-continuum, by projecting out the 2e bound states at each time step

$$\Psi_{1\text{b/}1\text{c}}\left(x,y,R,t\right)=\Psi \left(x,y,R,t\right)-\overset{19}{\underset{n=0}{\sum }}\left(\iint \varphi_{n}^{2\text{e}}\left(x^{\prime },y^{\prime };R\right)\Psi \left(x^{\prime },y^{\prime },R,t\right)dx^{\prime }\;dy^{\prime }\right)\varphi_{n}^{2\text{e}}\left(x,y;R\right).$$ (19)

This procedure is computationally very demanding and, therefore, limited to the early few femtoseconds. A maximum of 20 2e bound states has shown to be sufficient to obtain convergence. The as such calculated wave function can be used to identify trends in the integrated asymmetry of the PES without having to propagate the full wave function for long times. This is achieved by integrating the bound/continuum wave function once over positive (fwd) and once over negative (bwd) *x* direction, yielding the asymmetry as:

$$n^{\text{fwd}}\left(t\right)=\int_{0}^{120\;\overset{{}^{\circ}}{A}}dx\iint \Theta \left(25\;\overset{{}^{\circ}}{A}-|y|\right){\left|\Psi_{1\text{b/}1\text{c}}\left(x,y,R,t\right)\right|}^{2}dy\;dR,$$ (20)

$$n^{\text{bwd}}\left(t\right)=\int_{-120\;\overset{{}^{\circ}}{A}}^{0}dx\iint \Theta \left(25\;\overset{{}^{\circ}}{A}-|y|\right){\left|\Psi_{1\text{b/}1\text{c}}\left(x,y,R,t\right)\right|}^{2}dy\;dR,$$ (21)

$$\tilde{A}\left(t\right)=\frac{n^{\text{fwd}}\left(t\right)-n^{\text{bwd}}\left(t\right)}{n^{\text{fwd}}\left(t\right)+n^{\text{bwd}}\left(t\right)}.$$ (22)

Hereby, the grid boundaries and grid points of the electronic grid where halved to reduce the computational costs. Because this procedure is only performed to investigate the very early attosecond dynamics, the shorter grid is sufficient to get an insight into the early ionisation dynamics. As will be seen, the asymmetry defined by Eq. 22 produces quantitatively similar results to the asymmetry given by Eq. 16.

## 3 Results

### 3.1 Laser-Free Dynamics

**FIGURE 2:**
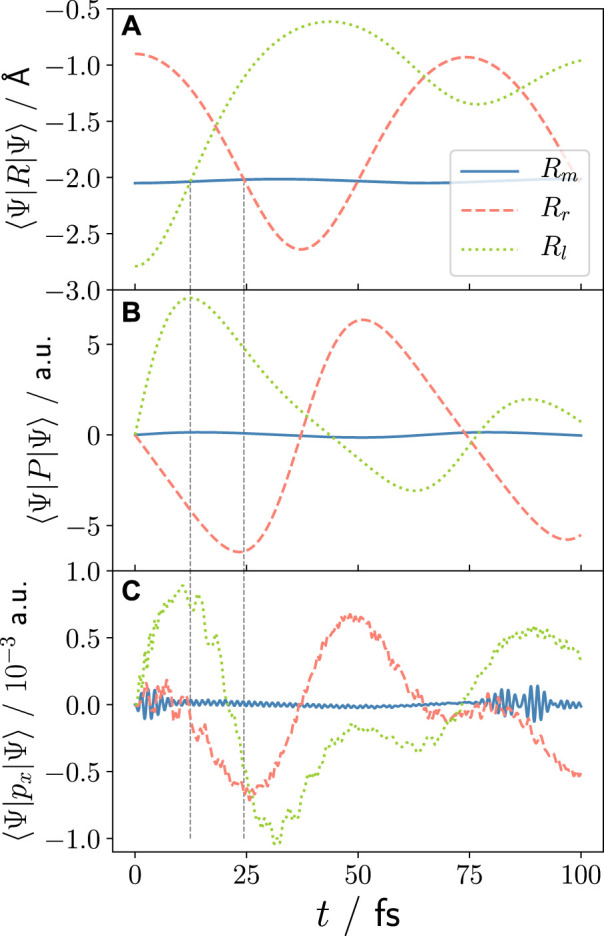
Laser-free dynamics in the 2e ground state for different initial wave functions (see Eq. 7): *R*_*m*_ for starting in equilibrium position *R*_0,*m*_ = −2.05 Å (blue), *R*_*l*_ and *R*_*r*_ for starting in non-equilibrium positions *R*_0,*l*_ = −2.79 Å (green) and *R*_0,*r*_ = −0.90 Å (red), respectively. **(A)** Nuclear coordinate expectation values, **(B)** nuclear momentum expectation values, and **(C)** electron momentum expectation values. The vertical dashed lines indicate the time when the nuclear wave packets reach the equilibrium position. This corresponds to the time of maximum nuclear and electron momentum, and the times around which the XUV pulse is centred in Subsection 3.2 and following.

In Figures 2A,B, we show the nuclear coordinate and momentum expectation values obtained for the three different initial nuclear wave packet starting positions *R*_0,*l*/*m*/*r*_. In 3), the corresponding mean electron momentum is displayed. Starting with the nuclear case, we see that for *R*_*r*_ an oscillating dynamics within the left potential well takes place (recall Figure 1B) with the nuclear momentum behaving accordingly. For *R*_*l*_, there is partial nuclear density transfer to the right potential well after approximately *t* > 25 fs, destroying the simple oscillatory motion of the nucleus. This is due to higher momenta being present in the nuclear wave packet that originate from the steep gradient left of the centre of *R*_0,*l*_. The electron response, i.e., the electron density’s momentum ⟨Ψ(*t*)|*p*_*x*_|Ψ(*t*)⟩, follows qualitatively the nuclear momentum, ⟨Ψ(*t*)|*P*|Ψ(*t*)⟩, however, with small discrepancies. For both non-equilibrium starting positions, the maximum nuclear and electron momentum expectation value is reached when the nucleus passes the minimum of the potential well, as indicated with the vertical, dashed lines in Figure 2.

### 3.2 Attosecond Dynamics

We aim at investigating the impact of nuclear motion and nuclear–electron correlation on the ultrafast ionisation dynamics. For this, we compare the ionisation process of the nuclear equilibrium configuration (*R*_0,*m*_) with ionisation of the initial non-equilibrium nuclear configuration (*R*_0,l/r_) with the pulse centred around the time of equilibrium passage, i.e., when ⟨*R*_l/r_(*t*_0_)⟩ = *R*_0,*m*_. These times are indicated previously in Figure 2 with the dashed vertical lines and correspond to a maximum positive (negative) nuclear and electron momentum for *R*_*l*_(*R*_*r*_). The times are *t*_0_ = 12.30 fs for *R*_l_(*t*_0_) = *R*_0,*m*_ and *t*_0_ = 24.405 fs for *R*_r_(*t*_0_) = *R*_0,*m*_. From now on, referring to *R*_*l*_ and *R*_*r*_ implicates this procedure, while *R*_0,l/r/m_ refers to the initial nuclear positions.

First, we analyse the attosecond dynamics during the XUV pulse interaction leading to the population of the parent ion states, $\varphi_{n}^{1\text{e}}\left(y;R\right)$ . Thus, we project the 1e states on the bound/continuum wave function

$$a_{n}^{1\text{e}}\left(t\right)=\iint {\left|\int \varphi_{n}^{1\text{e}}\left(y;R\right)\Psi_{1\text{b/}1\text{c}}\left(x,y,R,t\right)dy\;\right|}^{2}dx\;dR.$$ (23)

**FIGURE 3:**
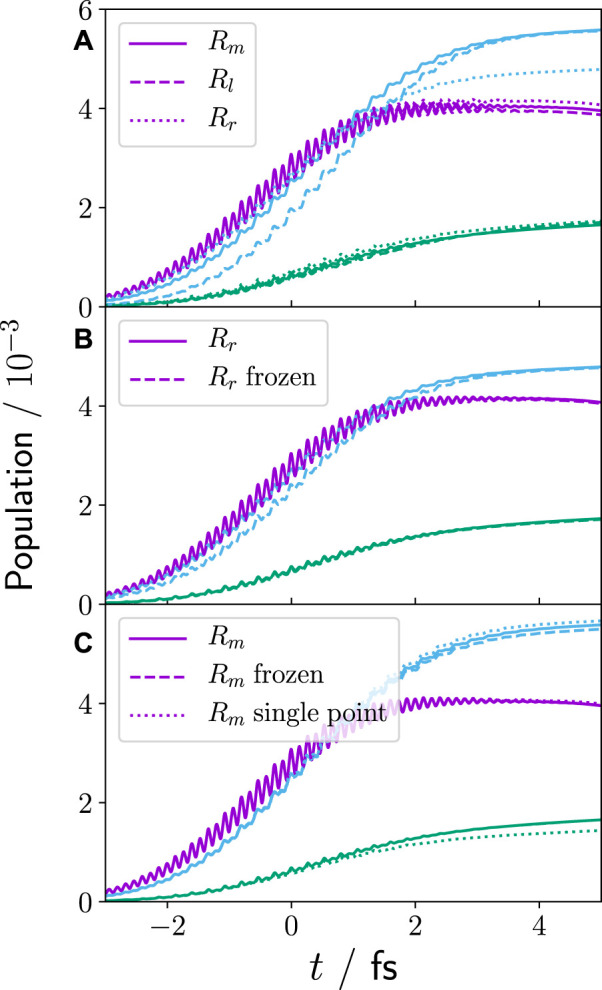
Population, $a_{n}^{1\text{e}}\left(t\right)$ , of the first three parent ion states, $\varphi_{n}^{1\text{e}}\left(y;R\right)$ (*n* = 0 (violet), *n* = 1 (green), *n* = 2 (blue)), during the XUV ionisation, Eq. 23. Panel **(A)** shows the comparison of equilibrium (solid lines), i.e., zero nuclear and electron momentum at time of ionisation, and non-equilibrium configuration (dashed/dotted lines), i.e., *R*_*l*_/*R*_*r*_: positive/negative nuclear and electron momentum at time of ionisation. **(B)** comparison of *R*_*r*_ and frozen nucleus *R*_*r*_, i.e., no quantum mechanical nuclear degree-of-freedom but sampled nuclear wave packet (see Subsection 2.1.2). **(C)** comparison of *R*_*m*_, frozen nucleus *R*_*m*_, and a single 2D purely electronic calculation with a point charge central nucleus at *R*_0,*m*_.

**FIGURE 4:**
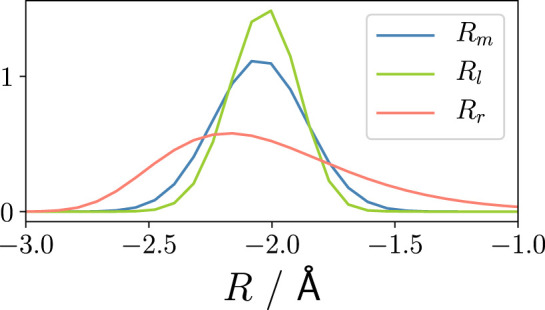
Nuclear wave packet at the time of ionisation, *t*_0_, for *R*_*m*_(*t*_0_), *R*_*l*_(*t*_0_), *R*_*r*_(*t*_0_), see text.

Figure 3A shows the population of the first three electronic parent ion states for the equilibrium and non-equilibrium cases (*R*_*l*_, *R*_*r*_). It can be seen that the previously discussed dynamics prior to ionisation, which leads to non-zero electron momentum at the time of ionisation, have almost no impact on the attosecond electronic population dynamics. The only small difference is visible for the *n* = 2 state in the case of *R*_*r*_ (blue dotted line). However, this difference does not originate from the nuclear–electron correlation but is rather due to the deformation of the nuclear wave packet prior to ionisation, see Figure 4, depicting the nuclear wave packet at the time of ionisation for *R*_*m*_(*t*_0_), *R*_*l*_(*t*_0_), and *R*_*r*_(*t*_0_). The deformation of the *R*_*r*_ nuclear wave packet, caused by the anharmonic PEC, leads to a small change in resonance conditions into the 1e-bound/1e-continuum and, consequently, to a slight change in the 1e state population. This becomes also evident by comparing the attosecond dynamics of *R*_*r*_ with a frozen nucleus calculation using the nuclear wave packet obtained at the time of ionisation from the 3D *R*_*r*_ simulation (recall Subsection 2.1.2, see Figure 3B). Although in the frozen nuclear wave packet simulation no nuclear or electron momentum is present, it shows identical attosecond dynamics. The frozen nuclear wave packet calculation without intrinsic quantum mechanical nuclear dimension reproduces the attosecond dynamics also for the other two investigated cases (*R*_*m*_, *R*_*r*_). More so, Figure 3C shows that for *R*_*m*_, a single 2D calculation with the classical point charge nucleus centred at −2.05 Å (the centre of the *R*_*m*_ wave packet) is sufficient to reproduce the attosecond dynamics and population of ionic states.

In other words, to describe the correct attosecond dynamics of the parent ion population upon ionisation, the quantum mechanical description of the nuclear degree of freedom can be neglected—a frozen nucleus approach of sampling the nuclear wave packet is sufficient. Moreover, if the nuclear dynamics prior to ionisation only proceeded on a harmonic PEC, leading to a compact Gaussian-like nuclear wave packet, a purely electronic TDSE simulation is able to reproduce the correct behaviour. This is an important finding for future approximations in the field of attosecond ionisation dynamics.

### 3.3 Momentum Conservation in Parent Ion Dynamics

**FIGURE 5:**
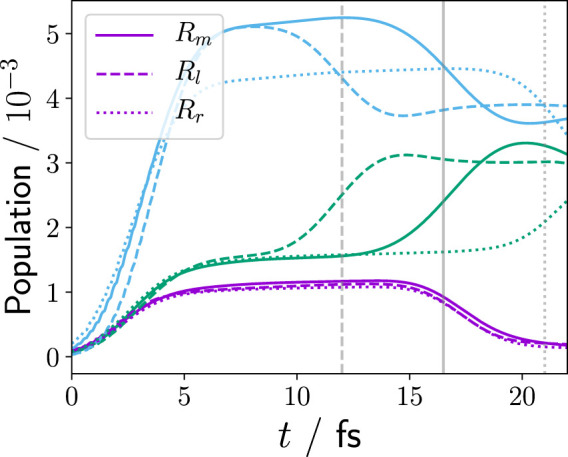
Population of parent ion 1e states using the bwd channel wave function (Eq. 24) to visualise the timing of the non-adiabatic transition between the *n* = 2 (blue) and *n* = 1 (green) state for *R*_*m*_ (solid lines), *R*_*l*_ (dashed lines), and *R*_*r*_ (dotted lines). The *n* = 0 population (violet) decreases as the channel wave function for this ion ground state with corresponding highest photoelectron momenta reaches the absorber after 15 fs. The difference in population of the *n* = 2 state for *R*_*r*_ compared to *R*_*m*_ is based on the nuclear wave packet deformation as discussed in the text for the bound/continuum wave function.

As seen in the previous section, within the first 4 fs upon XUV pulse interaction, the population in the parent ion is created and reaches stable values. Subsequently, in the parent ion, the nuclear wave packet moves on the corresponding PECs contained in the electronic wave packet acquiring momenta determined by the PEC’s gradients. In particular, the nuclear wave packet propagating on *n* = 2 and moving towards *R* = 0 undergoes a pronounced non-adiabatic transition with *n* = 1. Passing through *R* = 0 will lead to a change in the population of the parent ion states in the electronic wave packet. If ionisation into *n* = 2 and the subsequent nuclear relaxation would be independent of the initial nuclear momentum at *t*_0_, *P* ^2e^(*t*_0_), gained during propagation in the 2e electronic ground state (cf. Figure 2), the crossing at *R* = 0 would always be reached approximately 17fs after ionisation. However, if this initial nuclear momentum is retained upon ionisation, this will become visible through the timing of the non-adiabatic transition. Thus, the time at which the non-adiabatic crossing occurs is a direct measurement for pre-ionisation momentum dynamics. Figure 5 shows the parent ion state-resolved population obtained in bwd direction using the channel wave function

$$b_{n}^{1\text{e}}\left(t\right)=\iint {\left|\int \varphi_{n}^{1\text{e}}\left(y;R\right)\Psi_{\text{ch}}^{\text{bwd}}\left(x,y,R,t\right)dy\;\right|}^{2}dx\;dR$$ (24)

for *R*_*m*_, *R*_*l*_, and *R*_*r*_. The non-adiabatic transition leading to depopulation of *n* = 2 and population of *n* = 1 is clearly visible for all three cases. Moreover, the time of the transition is shifted to earlier (later) times for *R*_*l*_ (*R*_*r*_) demonstrating that the pre-ionisation momentum acquired by the nuclear wave packet propagating in the electronic ground state is retained upon ionisation. This is even more evident in Table 1 where the nuclear momentum expectation value of the individual nuclear wave packet propagating on one of the first three parent ion states is shown at *t* = *t*_0_ + 2.5 fs for the three initial starting positions. This is calculated by projecting bwd channel wave function on the corresponding ionic state and calculating the momentum expectation value:

$$\Psi_{\text{ch, n}}^{\text{bwd, }1\text{e}}\left(x,R,t\right)=\int \varphi_{n}^{1\text{e}}\left(y;R\right)\Psi_{\text{ch}}^{\text{bwd}}\left(x,y,R,t\right)dy,$$ (25)

$$P_{n}^{1\text{e}}\left(t\right)=\iint \Psi_{\text{ch, n}}^{\text{bwd, }1\text{e}\;*}\left(x,R,t\right)\;P\;\Psi_{\text{ch, n}}^{\text{bwd, }1\text{e}}\left(x,R,t\right)dR\;dx.$$ (26)

**TABLE 1:** Nuclear momentum expectation value of the different nuclear wave packets on different parent ion states, see Eq. 26, for the three different initial nuclear positions (first to third row). The fourth and fifth rows show the difference of non-equilibrium to equilibrium momentum, emphasising momentum conservation upon ionisation.

Starting position	*P*_0_^1e^(*t*_0_ + 2.5 fs)	*P*_1_^1e^(*t*_0_ + 2.5 fs)	*P*_2_^1e^(*t*_0_ + 2.5 fs)
*R*_*m*_	−0.24	2.92	5.42
*R*_*l*_	6.95	10.23	12.87
*R*_*r*_	−6.38	−3.57	−1.28
*R*_*l*_—*R*_*m*_	7.18	7.30	7.45
*R*_*r*_–*R*_*m*_	−6.15	−6.49	−6.70

It can be gathered from Table 1 that the nuclear momentum is different depending on the PEC the nuclear wave packet evolves on, e.g., for *R*_*m*_ (first row) the momentum is negative for *n* = 0, while it is positive for *n* = 1 and further increased for *n* = 2 as expected from the PEC gradients (Figure 1). Second, the momenta for the non-equilibrium cases *R*_*l*_ (*R*_*r*_) (second and third row) are uniformly shifted to higher (lower) momentum values. The lower rows, *R*_*l*_–*R*_*m*_ and *R*_*r*_–*R*_*m*_, quantify the difference to the equilibrium case (no initial momentum). These concur with the momentum in the bound 2e system at time of ionisation, which is for *R*_*l*_: *P* ^2e^(*t*_0_) = 7.6 a.u. and for *R*_*r*_: *P* ^2e^(*t*_0_) = −6.4 a.u (seen vertical dashed lines in Figure 2B). Therefore, we have unambiguously shown that the nuclear momentum in the bound 2e system is conserved upon ionisation manifesting itself in a change in timing for the non-adiabatic transition.

### 3.4 Photoelectron Spectra and Asymmetry

We now investigate to which extent these nuclear–electron correlation dynamics impact the PES. It has been reported that for simple one active electron systems, the coupled nuclear–electron dynamics are imprinted in the integrated photoelectron asymmetry (Falge et al., 2012a, 2017). So far, it is an open question, whether this also holds for multi-electron systems.

#### 3.4.1 Resonance Condition

**FIGURE 6:**
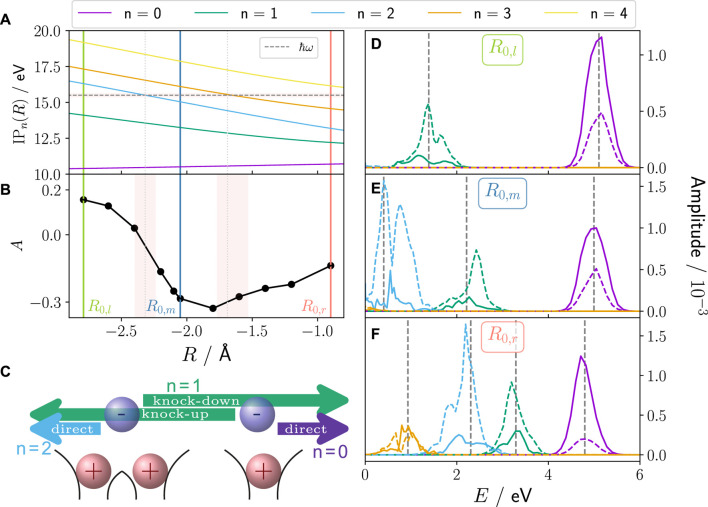
**(A)** Ionisation potential (Eq. 27) for different parent ion states and XUV pulse energy *ℏω* =15.5 eV (horizontal dashed line) with FWHM in light red. Vertical green, blue and red lines indicate the three initial nuclear positions, *R*_0,*m*_, *R*_0,*l*_, and *R*_0,*r*_, respectively. **(B)** Asymmetry is calculated with frozen nucleus simulation at different parametric *R* values (Eq. 16). **(C)** Scheme of the relevant ionisation pathways and the resulting ion state: direct ionisation of the weaker (stronger) bound electron dominant in fwd (bwd) direction leads to population of *n* = 0 (violet) (*n* = 2 (blue)) parent ion state; *n* = 1 parent ion state is populated via knock-up of weaker bound electron in bwd direction or via knock-down of stronger bound electron in fwd direction. Panels **(D)**, **(E)**, and **(F)** show the PES (solid lines: fwd, dashed lines: bwd) for frozen nucleus calculations at *R*_0,*m*_, *R*_0,*l*_, and *R*_0,*r*_, respectively. The dashed vertical lines indicate the expected peak position based on the resonance condition *E* = *ℏω* − IP_*n*_(*R*_0,*l*/*m*/*r*_).

The major difference to our previous work using single active electron systems is that upon ionisation, there is no ionisation into a single continuum state but rather complex transitions into several 1e-bound/1e-continuum states depending on the XUV pulse’s central frequency. Moreover, ionisation into these different states features varying resonance conditions for the various ion states. The resonance conditions are visualised in Figure 6A as a function of the parametric nuclear position

$${IP}_{n}\left(R\right)=V_{0}^{2\text{e}}\left(R\right)-V_{n}^{1\text{e}}\left(R\right).$$ (27)

For the three initial nuclear positions (*R*_0,*m*_, *R*_0,*l*_, *R*_0,*r*_), respectively, 3, 2, or 4 parent ion states are energetically accessible for the XUV pulse used in this work (*ℏω* = 15.5 eV). Figures 6D–F show the respective PES with exactly those 3, 2, or 4 peaks. Additionally, ionisation in these ionic states comes together with static and dynamic electron–electron correlation effects on top of any electron–nuclear correlation due to coupled dynamics. Therefore, depending on the position of the nuclear wave packet at the time of ionisation, there is a state-intrinsic inherent fwd/bwd asymmetry independent of any nuclear–electron coupling, originating from electron–electron interaction. Thus, each peak in the PES has a unique fwd/bwd asymmetry (see Figures 6D–F) leading to the overall photoelectron integrated asymmetry. The origin of the asymmetry for each peak is rooted in the different ionisation processes that lead to its population and are purely based on electron-electron interaction as reported in Fröbel et al. (2020) (see scheme in Figure 6C): *n* = 0 is predominantly populated via direct ionisation of the weaker bound electron (right of the central nucleus), which is favourable in fwd direction since it does not have to pass the other electron. *n* = 2 is the respective direct ionisation of the stronger bound electron (left of nucleus), which proceeds predominantly into bwd direction. *n* = 1 is in a bwd direction dominated knock-up ionisation process with smaller parts as knock-down process in fwd direction. As for different (parametric) *R* values, a different number of ionic states is accessible, with each of them featuring this intrinsic preference in the emission direction of the photoelectron, this leads to the overall parametric *R*-dependent asymmetry behaviour shown in Figure 6B. These results have been obtained by frozen nucleus calculations using Eq. 16 to calculate the asymmetry, thus, showing that it is a purely electron-electron correlation driven inherent asymmetry. Its *R*-dependency can be easily understood: starting from *R*_0,*m*_, the asymmetry rises for more negative *R* positions since the bwd-dominated *n* = 2 state becomes energetically inaccessible (Eq. 27). Equally, the overall asymmetry rises for larger *R* as the fwd/bwd neutral *n* = 3 state becomes energetically accessible. For a nuclear wave packet rather than a point-like *R*-value (see Figure 1) these two effects are smeared.

#### 3.4.2 Nuclear–Electron Correlation Imprint

**FIGURE 7:**
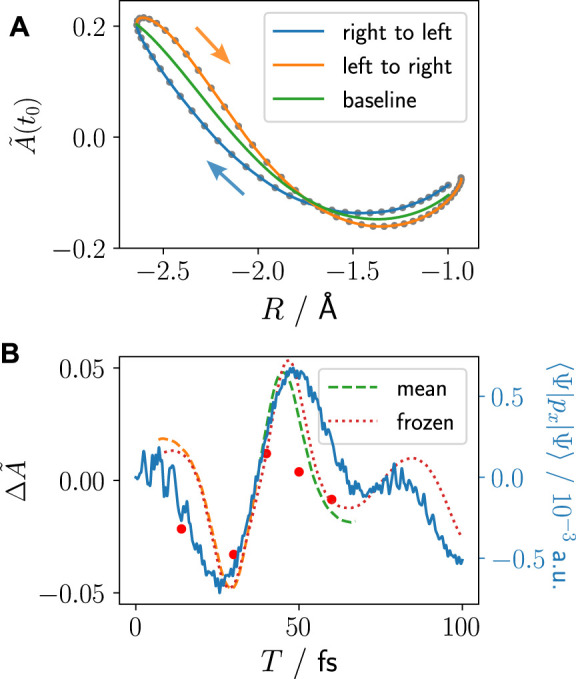
Imprint of nuclear–electron correlation in the asymmetry of the PES for a non-equilibrium starting position of *R*_0,*r*_ probed by a XUV pulse at different time-delays *T*: **(A)** Asymmetry calculated according to Eq. 22 at time of ionisation in dependence of the nuclear position *R* at time of ionisation, *t*_0_. Right to left and left to right refer to the time-delay intervals of *T* = [7,37]fs and *T* = [37,74]fs, respectively. The baseline is calculated using approach *1*) explained in the text as mean between left to right and right to left. **(B)** The nuclear–electron correlation-based asymmetry, $\Delta \tilde{A}$ , as a function of the time delay *T* obtained with the mean baseline (*1*, dashed) and the calculated frozen nucleus baseline (*2*, dotted). As explained in the text, the mean basline can only be obtained if forth and back propagation in the same potential is available (*T* = [7,37]fs and *T* = [37,74]fs), whereas the frozen nucleus baseline is available for all time delay points. The red dots stem from the asymmetry calculated with the more computationally demanding Eq. 16. The electron mean momentum is also shown, for comparison (solid blue line).

The procedure to visualise the imprint of pre-ionisation nuclear–electron correlation dynamics on the photoelectron is the following: we start at *R*_0,*r*_ and probe the integrated asymmetry by scanning the time delay of the XUV pulse interaction from *T* = [7, 100]fs in 1 fs interval steps. We start the integrated asymmetry calculation earliest at 7 fs to ensure sufficient time for the 5 fs broad XUV pulse. *R*_0,*r*_ was chosen as a starting point since the nuclear dynamics are constricted to the left potential well exhibiting a more distinct dynamics with larger imprinted momenta (see Figure 2). During the propagation, the nuclear wave packet propagates on the 2e electronic ground state PEC from the inner turning point *R*_0,*r*_ (right) to the outer turning point *R*_0,*l*_ (left) and back to *R*_0,*r*_, corresponding to the time-delay intervals of *T* = [7, 37]fs and *T* = [37, 74]fs, respectively (see Figures 1, 2). To save computational cost, we calculate the asymmetry from the bound/continuum wave function using Eq. 22, since it allows us to obtain converged asymmetries with fewer time steps and compare with some selected calculations using Eq. 16 that require propagation for long times. As is seen in Figure 6B, due to the changing resonance conditions, the inherent electron–electron correlation-based asymmetry varies for different parametric nuclear positions. The nuclear–electron correlation-based asymmetry is, thus, imprinted on top of the inherent electron–electron correlation-based asymmetry. Consequently, to extract the nuclear–electron correlation-based asymmetry, a “baseline” of the electron–electron correlation-based asymmetry during the nuclear propagation is required. This is carried out here in two approaches whose merits and shortcomings we will discuss shortly: *1*) a mean baseline is obtained from relating the asymmetry from forth and back movement of the nucleus (right to left vs left to right, Figure 7A). *2*) A baseline is calculated for each interval step using frozen nuclear wave packet calculations with the nucleus set to its position at the time of ionisation. Approach *1*) resembles more an experimental setup where a nuclear wave packet could be propagated forth and back in a potential, whereas a baseline by frozen nucleus calculations (approach *2*) cannot be obtained in the experimental setup, however, is not restricted to a forth and back movement of the nucleus in the same potential.

Figure 7A shows how the nuclear–electron correlation-based asymmetry is imprinted on top of the purely electron–electron correlation-based inherent asymmetry baseline that shows the same behaviour as shown in Figure 6B. The right to left (blue) and left to right (orange) curves are obtained by relating the corresponding time intervals *T* = [7, 37]fs and *T* = [37, 74]fs to the *R* value at the time of ionisation. In Figure 7B, the difference of the asymmetry to the two baselines 1*)* mean (dashed) and *2*) frozen (dotted) is shown with respect to the time-delay *T*. Comparison with the electron density’s momentum in the 2e bound system (solid blue line) shows very good agreement.

This means that starting the nucleus in a non-equilibrium position, which experimentally could be realised, e.g., by a first pump pulse, leads to coupled nuclear–electron bound dynamics that are imprinted on the electron density’s momentum. That, in turn, is imprinted on the photoelectron spectrum. Thus, we have demonstrated here that imprints of both, nuclear–electron and electron–electron correlated dynamics, are visible in the photoelectron spectra and the asymmetry of the photoelectron emission direction. We have generalised previous work and analysis on single-electron systems for the here investigated correlated system involving two electrons.

## 4 Summary

We set out to answer questions of particle correlation in molecular XUV ionisation and shed light on how these effects manifest in observables. To this end, we have employed a fully correlated molecular quantum model system comprised of two active electrons and one active nucleus that mimics a generic molecular system and allows us to report qualitative effects.

First, we examine what a coupled electronic–nuclear motion looks like in the bound system and showed the adiabatic imprint of the nuclear motion on the electronic momentum. Next, we focused on the implication of these coupled dynamics on the molecular XUV ionisation process, in particular to answer the question regarding characteristics in the attosecond population dynamics and post-ionisation dynamics. We report that the nuclear momentum is conserved during the XUV ionisation from the target to the parent ion and impacts the post-ionisation dynamics. On the other hand, the attosecond electron population dynamics are largely unaffected by the coupled nuclear–electron dynamics in the bound system. Finally, we turned towards features appearing in the photoelectron spectra and their relation to electron–electron and electron–nuclear correlation. The results drastically show how all particles are strongly correlated and imprint each other’s properties. Each photoelectron peak has an inherent asymmetry rooted in its electron–electron correlation-based ionisation pathways with its bound counterpart. The underlying complex bound/continuum resonances change when the nucleus is displaced leading to a change in electron–electron correlation-based photoelectron properties. On top of this, the initial coupled nuclear–electron momentum in the bound system is imprinted on the entangled photoelectron via its spectrum’s asymmetry and could be used as an experimental observable. While we have seen that correlation impacts pre, during, and post ionisation in various ways, well-designed approximations can be introduced at different stages depending on the intended outcome of the simulation. For example, the attosecond population dynamics are only influenced by a deformation of the nuclear wave packet pre-ionisation, which is not based on nuclear–electron correlation and can be reproduced by a sampling approach to the nuclear wave packet. In the case of harmonic PECs yielding a compact Gaussian-like nuclear wave packet, the nuclear degree of freedom can be safely ignored and a purely electronic description of the system with single-point nuclei reproduces the correct electronic parent ion wave packet population. Moreover, the momentum conservation in the nuclei between target and parent ion can be incorporated into classical approximations. Combining these ideas will be the subject of future research. The photoelectron asymmetries are purely based on the correlation effects of all particles and, thus, can only be observed when treating all particles quantum dynamically and with the corresponding exact correlation. However, approximated methods could be used to calculate the effect that is imprinted in the photoelectron asymmetry rather than the full correlated XUV ionisation process. For example, approximate theories can calculate the change in continuum resonances that lead to a change in the electron–electron correlation-based asymmetry, or the nuclear dynamics in the bound state that are imprinted on the spectrum via nuclear–electron correlation.

We are confident that our findings not only widen our understanding of fundamental correlation-driven processes in XUV ionisation but will also guide future experiments and approximated theory towards which effects have to be taken into account to properly describe correlation in molecular ionisation.

## Data Availability

The raw data supporting the conclusion of this article will be made available by the authors, without undue reservation.
